# Association between Suicide Ideation and Attempts and Being an Immigrant among Adolescents, and the Role of Socioeconomic Factors and School, Behavior, and Health-Related Difficulties

**DOI:** 10.3390/ijerph13111070

**Published:** 2016-11-01

**Authors:** Kénora Chau, Bernard Kabuth, Nearkasen Chau

**Affiliations:** 1 Département de Médecine Générale, Faculté de Médecine, Université de Lorraine, 9 Avenue de la Forêt de Haye, CS50184, Vandoeuvre-lès-Nancy F-54505, France; 2 INSERM Centre d’Investigations Cliniques Plurithématique 1433, UMR 1116, CHU de Nancy, Vandoeuvre-lès-Nancy F-54511, France; 3 Service de Pédopsychiatrie, Faculté de Médecine, Université de Lorraine, Hôpital d’Enfants de Nancy-Brabois, Vandoeuvre-lès-Nancy F-54500, France; kabuth_b@yahoo.fr; 4 INSERM, U1178, Paris F-75014, France; nearkasen.chau@wanadoo.fr; 5 Univ Paris-Sud, UMR-S1178, Paris F-75014, France; 6 Univ Paris Descartes, UMR-S1178, Paris F-75014, France

**Keywords:** immigrant adolescents, suicide behaviors, socioeconomic factors, school, behavior, health difficulties, risk factors

## Abstract

The risk of suicide behaviors in immigrant adolescents varies across countries and remains partly understood. We conducted a study in France to examine immigrant adolescents’ likelihood of experiencing suicide ideation in the last 12 months (SI) and lifetime suicide attempts (SA) compared with their native counterparts, and the contribution of socioeconomic factors and school, behavior, and health-related difficulties. Questionnaires were completed by 1559 middle-school adolescents from north-eastern France including various risk factors, SI, SA, and their first occurrence over adolescent’s life course (except SI). Data were analyzed using logistic regression models for SI and Cox regression models for SA (retaining only school, behavior, and health-related difficulties that started before SA). Immigrant adolescents had a two-time higher risk of SI and SA than their native counterparts. Using nested models, the excess SI risk was highly explained by socioeconomic factors (27%) and additional school, behavior, and health-related difficulties (24%) but remained significant. The excess SA risk was more highly explained by these issues (40% and 85%, respectively) and became non-significant. These findings demonstrate the risk patterns of SI and SA and the prominent confounding roles of socioeconomic factors and school, behavior, and health-related difficulties. They may be provided to policy makers, schools, carers, and various organizations interested in immigrant, adolescent, and suicide-behavior problems.

## 1. Introduction

Every year, over 800,000 people die by suicide worldwide [1]. Suicide is the second leading cause of death among the subjects aged 15–29 year [1]. The prevalences of suicide ideation in the last 12 months (SI) and lifetime suicide attempt (SA) were respectively 9.6% and 8.9% in France, and 16.8% and 7.6% in the United States [2]. Most transitions from suicide ideation to suicide attempt (60%) occur within one year [3]. It is estimated that 15%–23% of people having consulted a physician for a suicide attempt will relapse in the year [4], and 5%–10% will die from suicide during the five following years [5]. According to the World Health Organization, there are multiple contributing factors (financial difficulties, discrimination, a sense of isolation, violence, substance use, mental disorders, etc.), and multiple vulnerable groups, especially refugees and immigrants [1]. Consequently, selective prevention strategies to assist vulnerable individuals are needed [1]. It is thus important to identify the risk patterns of SI and SA and to evaluate the confounding roles of potential covariates in younger immigrant adolescents (10–16 years) who may be a vulnerable group. With increasing immigration, European Union health systems face new challenges in terms of rights to health, access to care, and health monitoring.

However, immigrants are from many countries and have different reasons (poverty, war, trauma, political repression, etc.) and histories for immigration. They have different educational, socioeconomic, occupational, and cultural characteristics. The integration opportunities may vary across host countries. Immigrants may meet difficulties in terms of language, socioeconomic, employment, and cultural integrations as well as physical and mental health, substance use and cares [6]. However, certain immigrants may have strong family networks or protective cultural or religious traditions [7]. Immigrant suicide behaviors are a complex problem [6]. The literature is abundant for adults but rather scarce for adolescents. According to a review of literature, little research has focused on suicide behaviors in immigrant youth, most research does not differentiate ethnic minorities, and the risk of suicide behaviors varies by ethnicity and country of settlement [8]. The available studies have reported that various immigrant groups can be at an increased or lower risk of suicide behaviors than the majority population [6,7,9]. A study in the Netherlands found that the rates of suicide attempts among Turkish and South Asian-Surinamese female adolescents were higher than that among their Dutch counterparts but Moroccan females had a lower rate [10]; this study also reported that that physical and sexual abuse and an impaired family environment contributed to non-fatal suicide behavior of females across ethnicities but these factors as well as low socioeconomic class and educational level did not fully explain the risk in Turkish and South Asian-Surinamese females [10]. A study in the United States reported that Korean immigrant adolescents had a higher risk of suicide ideation than their American counterparts and Koreans who remained in Korea, and the risk was associated with life stress, lack of parental support, and not living with both parents [11]. Another study in United States showed that US-born Latinos with immigrant parents and those with U.S.-born parents had a three-fold higher risk of suicide attempt [12]. In Canada, the suicide rate was low for immigrants considered as a whole, but various national origin groups reported different trajectories across the generations [13]. The risk of suicide behaviors in immigrant adolescents remains thus an open question.

Early adolescence is an important period for physical and mental development [2,14,15]. It is a period of transition from the total social and economic dependence to relative independence with more contacts and exchanges with others and more access to substance use. Unfortunately, it corresponds to the mean age of onset of school, behavior, and health-related difficulties (such as grade repetition, poor health, depressive symptoms, substance use, and violence) [2,16,17,18,19]. A recent prospective study examining developmental trajectories of suicide ideation across early to middle adolescences found that the highest risk was at age 12 for boys and age 12–13 for girls, and that depression, externalizing problems, and family and friend support played a role for the two sexes [20]. Immigrant adolescents furthermore face to isolation, acculturation related stressors, ethnic discrimination, and social and school integration [16,21]. They have more frequently poor parents’ education, non-intact families, poor socioeconomic resources, economic uncertainty, and access to health care [16,21,22,23,24,25]. They also have more school, behavior, and health-related difficulties [16,26,27]. These sets of factors may predict suicide behaviors [2,28]. School, behavior, and health-related difficulties could alter physical and mental performances and cognitive development [29,30,31,32,33,34]. Therefore, immigrant adolescents may have a higher risk of SI and SA from an early age. A better knowledge of the role of socioeconomic factors and school, behavior, and health-related difficulties in SI and SA may help when designing prevention. Suicide is largely preventable [1] and most of these difficulties are modifiable for prevention. The role of these problems among immigrant adolescents has remained insufficiently addressed [27].

In an early adolescence context and using historic reconstruction of life events, this study in France assessed the risk of SI and SA of immigrant adolescents compared with their native counterparts, and the confounding role of socioeconomic factors as well as school, behavior, and health-related difficulties. We focused on middle-school adolescents mostly under 16 years because school is compulsory until 16 years, many problems become persistent in late adolescence (16–20 years) and all issues should be solved at an early age [2,35] via screening and monitoring.

## 2. Materials and Methods

### 2.1. Study Design

The study population comprised all 1666 students attending three middle schools (age range 10–19, two public and one private (63 classes)), chosen as it may reflect a social gradient (various social categories are represented) in the Nancy urban area (410,000 inhabitants), the capital of Lorraine region (2.34 million inhabitants) in north-eastern France. The study population represented the exhaustive population of a large geographical area (38,000 inhabitants). The investigation was approved by the Commission Nationale de l’Informatique et des Libertés (national review board, Project 1408688) and the Nancy-Metz regional education authority. Written informed consent was obtained from parents. In contrast to previous national studies [2,14,36] we focused on a population from an urban area so that the subjects were in the same socioeconomic context, free of variations across geographical regions.

The study protocol included an invitation to participate transmitted to parents and guardians (April 2010) and data were collected (May–June 2010) using an anonymous self-administered questionnaire over a one-hour teaching period under research-team supervision. Respondents could ask the two research-team members if they did not understand a question, but team members had been instructed not to influence the response (the adolescents rarely asked questions). Adolescents placed the completed questionnaires in a sealed envelope and then into a closed box. Two students refused to participate and 89 (5.3%) were absent when the data collection was carried out (for motives independent of the survey). In total, 1575 adolescents completed the questionnaires; among which 10 were of unknown gender or age, and 6 were not completed appropriately, leaving 1559 questionnaires (94%) for analysis. This population was close to that of a French school-based population survey in terms of gender, family and health-related difficulties (Table 1).

The questionnaire included age, gender, socioeconomic characteristics (nationality, family structure, father’s and mother’s education, father’s occupation, and family income), grade repetition, behavior and health-related difficulties (alcohol, tobacco, cannabis and hard-drug use, depressive symptoms, sustained physical and verbal violence, and sustained sexual abuse), SI, and SA [2,14].

All subjects gave their informed consent for inclusion before they participated in the study.

### 2.2. Measures

#### 2.2.1. Suicide Ideation and Suicide Attempts

Suicide ideation (SI) was addressed in the question “During the last 12 months, did you ever think about suicide?” (response: any/none). Suicide attempts (SA) was assessed in the question “During your life course, how many times did you actually attempt suicide?” (response: none, 1, or 2 or more) [2,14]. SA was defined as at least once.

#### 2.2.2. Family Structure

Three categories were considered: (a) intact families corresponded to the adolescents who were living with both non-separated and non-divorced father and mother; (b) divorced or separated parents and reconstructed families corresponded to the adolescents who had parents separated or divorced with the presence or not of a father- or a mother-in-law; and (c) single parent and other situations. Among the 391 subjects who were living with divorced or separated parents or reconstructed families, 82.9%, 9.0%, 4.6%, and 3.6% were respectively living generally, sometimes, rarely, and never with mother (respectively 39.4%, 32.0%, 17.1%, and 11.5% with father). Because of a small number of subjects which would result in a lack of power for statistical tests, the time spent with father and mother was not considered.

#### 2.2.3. Father’s Occupation

Five categories were considered following the international classification of occupations (ISCO): managers, professionals, and intermediate professionals (reference group); craftsmen, tradesmen, and heads of firms; service workers and clerks; manual workers and other occupations; and non-working people (unemployed and retired) [36,38,39].

#### 2.2.4. Grade Repetition

Grade repetition was assessed with the question “During your life course, do you have repeated school year(s) at primary school and middle school?” (response: never, at primary school, for every year at middle school); multiple responses were possible [16]. The year of grade repetition(s) was gathered.

#### 2.2.5. Risky Behaviors (Alcohol, Tobacco, Cannabis, and Hard-Drug Use)

For each substance, we retained only the lifetime use that had continued until the time of survey (current use). Alcohol initiation was assessed with the question: “When (if ever) did you first drink (beer, cider, champagne, wine, aperitif, etc.)?”; tobacco initiation with the question: “When (if ever) did you first smoke your first cigarette?”; cannabis initiation with the question: “When (if ever) did you first try any form of cannabis?”; and initiation of other illicit drugs with the question: “When (if ever) did you first try any form of other illicit drugs (mushrooms, ecstasy, LSD, etc.)?” (response: year of initiation) [2,14,19,36]. The year of use initiation of each substance category was thus gathered.

Current use of alcohol was assessed with the question: “During the last 30 days, how many times have you had alcoholic drinks (beer, cider, champagne, wine, aperitif, etc.)?” (response: none/1–5/≥6); that of tobacco with the question: “During the last 30 days, how many cigarettes a day did you smoke?” (response: none/≥1 cigarette per day); that of cannabis with the question: “During the last 30 days, how many occasions have you used any form of cannabis?” (response: none/≥1); and that of other illicit drugs with the question: “During the last 30 days, how many occasions have you used any form of other illicit drugs (mushrooms, ecstasy, LSD, etc.)?” (response: none/≥1) [2,6,11,28].

#### 2.2.6. Depressive Symptoms

Depressive symptoms were evaluated using the six-item Kandel scale [2,40]. Participants were asked to indicate the degree to which they (a) “felt too tired to do things”; (b) “had trouble going to sleep or staying asleep”; (c) “felt depressed”; (d) “felt hopeless about the future”; (e) “felt nervous or tense”; and (f) “worried too much about things”. Participants responded to each item using a four-point Likert scale ranging from 1 = never, 2 = rarely, 3 = often enough to 4 = very often. This scale is sex and longitudinal invariant and useful in research exploring psychological distress throughout adolescence [41]. The starting year over the life course was gathered. The Cronbach’s alpha was satisfactory (0.84) allowing a single score to be calculated (range 6–18). Depressive symptoms were defined by a score ≥17 (90th percentile value) [2,42]. Note that 95% of subjects actually reported feeling depressed.

#### 2.2.7. Physical and Verbal Violence

Physical and verbal violence sustained by adolescent was measured using a 20-item scale (five questions for four localities): “During the last 12 months, have you been victim of …?”: (1) hitting; (2) stealing; (3) racket; (4) insult; and (5) racial abuse; in (a) school; (b) school neighborhood; (c) at home; and (d) elsewhere (response: any/none) [2,14]. The Cronbach’s alpha was satisfactory (0.71), allowing a single score to be calculated as the number of positive responses. Sustained violence was defined by a score ≥4 (90th percentile). The year of the first occurrence over the life course was gathered.

#### 2.2.8. Sexual Abuse

Sexual abuse sustained was assessed with the question: “In the course of your life, have you been victim of a sexual abuse?” (response: any/none) [2,14]. The year of the first occurrence over the life course was gathered.

#### 2.2.9. Historic Reconstruction of Life Events

A historic reconstruction of life events from birth to the day of survey was made using retrospective data gathered. During the observation period which represented 14,530 person-years, 154 SA were observed (135 and 19 subjects among French and immigrant adolescents, respectively). SI affected 182 subjects (158 and 24 adolescents, respectively).

### 2.3. Statistical Analysis

First, we compared European immigrant, non-European immigrant, and French adolescents according to SI, SA, socioeconomic factors and school, behavior, and health-related difficulties using the chi^2^ test or the variance analysis. Then, for SI, we assessed its association with being European or non-European immigrant and various risk factors using gender-age-adjusted odds ratio (gaOR) computed with logistic regression models. Because results showed that European and non-European immigrant adolescents had similar features, they were grouped to avoid a lack of power for statistical tests. To evaluate the confounding roles of socioeconomic factors and school, behavior, and health-related difficulties in the risk of SI for immigrant adolescents, three logistic regression models were performed: a basic model (model 1) measuring the association between SI and being immigrant after adjustment for gender and age, then socioeconomic factors added to model 1 (model 2), and finally school, behavior and health-related difficulties added to model 2 (model 3). The contribution of each set of covariates to the explanation of the association between SI and being immigrant was estimated by the change in the odds ratio after its inclusion in the model, i.e., explained fraction calculated by the formula: (OR_model1_ − OR_extended model_)/(OR_model1_ − 1) [43]. Similar analyses were made for SA but using Cox regression models to compute hazard ratios (HR). In these Cox regression models, various school, behavior, and health-related difficulties were considered only when they started before SA. All the analyses were performed using the Stata program (Stata Corporation, College Station, TX, USA, 2007).

## 3. Results

### 3.1. Characteristics of the Study Population

The characteristics of European immigrant, non-European immigrant and French adolescents are shown in Table 2. SI affected more European and non-European immigrant adolescents than their native counterparts (20.4% and 24.1% vs. 10.9%, *p* = 0.002). Similar differences were found for SA (18.5% and 16.7% vs. 9.3%, *p* = 0.02). The crude rate of SA per 1000 person-years was 20.7 for European immigrant adolescents, 17.8 for non-European immigrant adolescents, vs. 10.0 for French adolescents. Multiple suicide attempts (≥2) also affected more European and non-European immigrant adolescents (9.3% and 11.1% vs. 4.3% for French adolescents, *p* = 0.02). Compared with French adolescents, immigrant adolescents had much more frequently non-intact families, lower parents’ education, lower father’s occupation, insufficient family income, grade repetition, and tobacco, cannabis, and hard-drug use. Sustained sexual abuse was more frequent among non-European immigrants.

**Figure 1:**
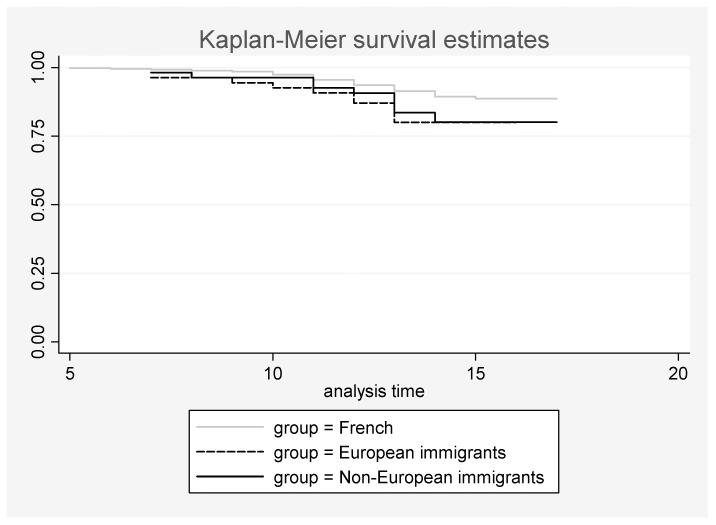
Frequency of French, European-immigrant, and non-European-immigrant adolescents with no suicide attempt according to age (year). The log-rank test for equality of the “survivor functions” (for suicide attempt) was significant with *p* = 0.016 (and with *p* = 0.005 when European and non-Europeans were grouped).

Figure 1 reveals that the risk of SA was similar enough for European and non-European immigrant adolescents and it started from an early age.

### 3.2. Associations of SI and SA with Socioeconomic Factors and School, Behavior, and Health-Related Difficulties—Contribution of These Covariates

Table 3 shows that, based on gaOR and gaHR, both European and non-European immigrant adolescents had a two-time higher risk of SI and SA. A higher risk of SI was observed for girls, the subjects living with divorced or separated parents or reconstructed families, those having a father being a manual worker or non-working, low parents’ education, insufficient family income, and various school, behavior and health-related difficulties (gaOR between 1.37 and 9.52). The risk factors for SA were female gender, living with divorced or separated parents or reconstructed families, living with single parents, non-working father, and various school, behavior and health-related difficulties (gaHR between 1.46 and 12).

Using nested logistic and Cox regression models, we found that after inclusion of socioeconomic factors in the first stage and of school, behavior and health-related difficulties in a second stage, the excess risk of SI found for immigrant adolescents was substantially reduced by 27% and 24% but it remained significant (Table 4). More importantly, we found that the excess risk of SA for immigrant adolescents was highly reduced by 40% and 85% and became non-significant (Table 4).

## 4. Discussion

This study found that immigrant adolescents had a two-time higher risk of both SI and SA, and that socioeconomic factors explained 27% and 40% of the risks while further considering school, behavior and health-related difficulties explained 24% and 85%, respectively leaving the SA risk become non-significant. As most transitions from suicide ideation to suicide attempt (60%) occur within one year [3], the high role of school, behavior and health-related difficulties in SI, and more especially in SA, is an important finding. These last factors are modifiable and may be targets for prevention policy aiming at reducing suicide attempt among immigrant adolescents. Our findings based on a historic reconstruction of life events for SA are an additional piece to the literature.

We focused on early adolescence because suicide behaviors are common in this age period [2,17] and prevention to reduce their risks should be performed since an early age. Many adolescents early suffer from a number of poor living conditions, socioeconomic deprivations and school, behavior, and health-related difficulties [2,14,17,18,19,36,37]. It may be noted that the mean age of parents’ divorce or separation is six years, that of parent’s death is eight years, and the first years at middle school correspond to the mean age of onset of substance use, sleep disorders, violence victimization, and suicidal ideation [2,14,17,18,19,36,37]. The risk of suicide behaviors may be higher when the issues begin at an early age. Indeed, two national adolescent studies in France and United States reported that early use initiation of alcohol, tobacco or cannabis (before 13 years) was associated with a higher risk of suicide behaviors [2]. One study in Korea found similar results [44]. A prospective study exploring the developmental trajectories of suicide ideation shows that the highest risk is at age 12–13 years, and that depression, externalizing problems, and family and friend support play a role [20].

Our study demonstrates that immigrant adolescents face an excess risk of SI and SA and this is highly attributable to socioeconomic factors (27% and 40%, respectively). We focused on immigrant adolescents who did not have the French nationality because they would be particularly vulnerable. A recent review of literature stated that first generation immigrant adolescents experience a higher rate of bullying and peer aggression compared to third generation and native adolescents [27]. In France, there were, in 2007, 2.56 million children aged less than 18 years in immigrant families. With increasing unemployment, parents of immigrant adolescents have more difficulty finding a job. Most immigrants were from developing countries with much lower resources [45]. Indeed, the birth country of the head of family of immigrant adolescents was in Africa (1.37 million children, mainly Algeria, Morocco, and Tunisia), Europe (642,302 children, mainly Portugal, Spain, Italia, and United Kingdom), Asia (393,775 children, mainly Turkey and Indochina), and America and Oceania (147,326 children) [46]. We found that immigrant adolescents were more likely to live in non-intact families. Divorced or separated parents and reconstructed families were more represented among European immigrants while single parents were more represented among non-European immigrants. European immigrants were more likely to have a father being a manual worker while non-European immigrants to have a non-working father. Low parents’ education and insufficient family income were also more represented among both European and non- European immigrant adolescents. It is rather difficult, especially for immigrants, to find a job in France as it has a high unemployment rate (10.2%) compared with some western Europe countries (Germany 4.2%, Great Britain 4.8%, The Netherlands 5.8%, Austria 6.2%, Luxembourg 6.2%, Sweden 7%, and Belgium 8.2%) [47]. In France, it is also hard to find an affordable and satisfactory accommodation for many families as social rental housings are lacking and 3.8 million people have poor housings. These social and material deprivations generally started at an early age and result in many adolescents face a number of school, behavior, and health-related problems. It may be noted that, in our study, strong associations were found between socioeconomic factors and various school, behavior, and health-related difficulties (results not shown). While research has showed that certain immigrant groups have a lower risk of suicide behaviors than the majority population [6,7,9], our results agreed with certain studies which have found that immigrant adolescents had a higher risk and that poor family environment, lack of parental support, not living with both parents, substance use, physical and sexual abuse, and poor mental health play strong confounding roles [6,10,11,20,48,49]. Some studies reported that the risk of suicide ideation was higher among adolescents who have family problems or difficulties in relationships with parents [6,8,9,11,48,49]. The children of immigrants often reach a higher level of acculturation and education than their parents, contributing to intergenerational conflict, decreased understanding, and closeness between children and parents [6,50].

Our study found that school, behavior, and health-related difficulties (added to socioeconomic factors) highly explained the excess risk of SA for immigrant adolescents (85%) which became non-significant. In the literature, one study in the United States reported that Korean immigrant adolescents had a higher risk of suicide ideation in which life stress played a role [11]. We found that school, behavior, and health-related difficulties affected much more immigrant adolescents than their native counterparts. School difficulty may reflect somewhat a mental and cognitive ability. Immigrant adolescents with school difficulty may see school as uninteresting, unchallenging, overwhelming or non-supportive. This situation may lead them to develop negative attitudes [51], to avoid school and all it represents, and finally to mental vulnerability favoring SA. Depressive symptoms are well known to affect memory, cognitive ability, executive functions, and work performance [29,42,52,53,54]. Tobacco and alcohol use could alter physical function, psychomotor function, and cognitive performance [31,32,55,56]. Cannabis use could exacerbate mental health difficulties [33]. Regarding sexual abuse and physical and verbal violence, research has showed that they generate substance use, stress, depressive, and internalizing symptoms, hopelessness, child adaptation failure, and damage to cognitive development [30,34,57,58] as well as suicide behaviors [2,10,28]. Children who have poor family functioning, family conflicts, or see their peers as hostile have a higher risk of suicide ideation [6,8,9,11,48,49]. So, various difficulties may play concurrent roles over time leading to long lasting mental vulnerability and consequently to SI and SA. Suicide results often from a difficult life trajectory that needs to be socially and medically monitored. One study reported the suicide of an adolescent aged 16 who committed a suicide attempt after the death of his father, followed by a depression, increasing alcohol use, sustained violence, and involvement in rioting at school [59]. Public policy aiming at reducing suicide ideation, and especially suicide attempts, should include services and interventions to solve school, behavior, and health-related difficulties. Further research may focus on these interventions and their evaluation using a prospective approach. It should be indicated that health problems of people with social and material deprivations are less likely to be treated [60,61,62]. In France, many people are under the poverty threshold, especially in single-parent and inactive-mother families [63] and do not have a complementary health insurance [64]. These issues may affect many more immigrants than native adolescents. Finally, public policy, schools, and school medical centers may evaluate and identify the immigrant students with higher socioeconomic difficulties and higher school, behavior, and health-related difficulties to help them to reduce their problems with the collaboration of general practitioners (carers most consulted by adolescents for their physical health, mental health, and substance use [17]), other carers, social workers, and their parents. However, it should be noted that counselling services, psychotherapeutic institutions, and schools are seldom sufficiently prepared or equipped for dealing with children and families’ concerns [65]. Education on mental health for immigrants and education about cultural differences for specialists are important for the advancement of suicide prevention [66].

### Limitations and Strengths

First, the study was cross-sectional and based on self-reported data, but a self-administered anonymous questionnaire is widely used and arguably a good tool to study the living conditions, mental health, and risky behaviors of adolescents [2,14,16,36,67]. The questionnaire was simple and would not be influenced by adolescent’s intellectual performance. Adolescents generally know the socioeconomic situations of their family, especially the financial difficulty. A study on family factors and substance use among adolescents showed that self-report data were corroborated by independent teacher reports [68]. Father’s occupation is generally known by the adolescents even in those who were living with single parents. The immigrant students were well distributed in various classes. The public school for each student was determined by his/her residence place and the class was attributed by the school. In France, we cannot investigate specific ethnic groups (national review board). We focused our survey on the population from an urban area so that the subjects are in the same socioeconomic context, free of variations across geographical regions. Given the large number of statistical tests carried out, type I error may be a concern, but most tests were significant at the 0.001 level, with very high odds ratios estimates.

Strengths of the study also deserve to be mentioned. The participation rate was high. The data collection was made under the research-team supervision with no influence on the survey. The statistical approach using Cox regression model may explore causal relationships for SA. However, these were not guaranteed because certain life events may be forgotten, but they were relatively recent and the adolescents affected would well remember them [69]. All were made to guarantee the respondents’ anonymity. For this purpose, the questionnaire excluded the birthday, the birth place, and the residential town. Data collected and the respondents’ identification number do not allow the determination of school and the precise class. The quality of responses to the questionnaire was good. Various instruments were used in adolescent studies in many countries [2,14,37,40]. The behavior and health-related difficulties of the sample were similar to those of France.

## 5. Conclusions

This study demonstrates that suicide ideation and attempts affect two-time more immigrant adolescents than their native counterparts and they are strongly associated with socioeconomic factors and school, behavior, and health-related difficulties. Socioeconomic factors play a strong confounding role (contribution 27% to the excess risk of suicide ideation and 40% of that of suicide attempts). Further considering school, behavior, and health-related difficulties explained 24% of the excess risk of suicide ideation and 85% of that of suicide attempts which became then non-significant. Our findings may be provided to policy makers, schools, carers, and other organizations interested in adolescent, health, and immigrant problems. They demonstrate that, to reduce suicide ideation, and particularly suicide attempts, public policies should identify, monitor, and reduce school, behavior, and health-related problems. Schools and school medical centers have an important role in school-based prevention programs. General practitioners and other carers have a prominent role and should cooperate with schools and school medical centers. Our findings may be given to parents to make them more aware of the problems, to improve adolescents’ learning and living conditions, to adopt mindful parenting [70], and to suggest them to identify and reduce school, behavior, and health-related issues with the help of school, school medical centers and carers. The adolescents should be helped to be aware of the problems, to adopt protective attitudes, to increase resilience [71], and to solve their problems with their parents, schools, school medical centers, and carers.

## Figures and Tables

**Table 1:** Comparison between the study population and France [2,12,37]: %.

Characteristics of Subjects	Study Population	France (ESPAD Survey)
	(Limited to <16 Years ^a^)	<16 Years
*Number of subjects*	*1524*	*8367*
Suicide ideation (last 12 months)	11.6	9.1
Lifetime suicide attempt	9.6	7.2
Girls	50.1	51.1
Family structure		
Intact	63.2	74.7
Reconstructed	15.0	11.3
Single parent	16.4	11.7
Others	5.4	2.3
Obese (with self-reported data)	10.6	6.9
Substance use (last 30 days)		
Tobacco	10.7	13.6
Alcohol	34.7	34.6
Cannabis	5.1	5.5
Sleep disorders	32.6	29.0
Asthma	17.2	16.3
Depressive symptoms	13.1	9.8
Sexual abuse	3.4	1.9
Victim of physical and verbal violence (at least once)	53.3	51.5
Involvement in violence (at least once)	59.1	64.7

**^a^** were excluded 35 subjects aged 16 years or over.

**Table 2:** Associations between nationality and various factors: % or mean (SD).

Characteristics of Subjects	French Adolescents	European Immigrant Adolescents	Non-European Immigrant Adolescents	*p* Value
*Number of subjects*	*1451*	*54*	*54*	
*Number of person-years*	*13,541*	*483*	*506*	
Suicide ideation (during the last 12 months, SI)	10.9	20.4	24.1	0.002
Suicide attempt (during the life course)				
At least one (SA)	9.3	18.5	16.7	0.020
Crude rate per 1000 person-years	10.0	20.7	17.8	
Two or more	4.3	9.3	11.1	0.018
Girls	50.0	55.6	48.2	0.692
Age (year)	13.0 (1.3)	12.8 (1.1)	13.4 (1.7)	0.021
Socioeconomic factors				
Family structure				<0.001
Intact	63.8	57.4	46.3	
Divorced or separated parents and reconstructed family	24.7	35.2	25.9	
Single parent and other types	11.5	7.4	27.8	
Father’s occupation				<0.001
Managers, professionals, and intermediate professionals	29.1	18.5	13.0	
Craftsmen, tradesmen, and firm heads	19.9	24.1	22.2	
Service workers and clerks	9.2	5.6	13.0	
Manual workers and other occupations	17.0	29.6	20.4	
Not working	6.9	9.3	22.2	
Low parents’ education (<university)	47.6	66.7	59.3	0.007
Insufficient family income	16.9	25.9	31.5	0.006
School, behavior, and health-related difficulties				
Grade repetition	13.6	22.2	37.0	<0.001
Alcohol consumption ^a^	35.6	31.5	29.6	0.564
Tobacco consumption ^a^	10.5	16.7	24.1	0.003
Cannabis consumption ^a^	5.1	9.3	14.8	0.005
Hard-drug consumption ^a^	2.3	7.4	11.1	<0.001
Depressive symptoms	13.1	18.5	14.8	0.489
Victim of physical and verbal violence	9.0	14.8	13.0	0.562
Victim of sexual abuse	3.4	1.8	11.1	0.010

**^a^** Was considered only the consumption which had continued until the day of survey.

**Table 3:** Factors associated with suicide ideation and suicide attempt: gender-age-adjusted odds ratio (gaOR) or hazard ratio (gaHR) and 95% confidence interval (CI).

Risk Factors	Suicide Ideation in the Last 12 Months (SI)		Lifetime Suicide Attempt (SA)	
	gaOR	95% CI	gaHR	95% CI
*Number of subjects*	*1451*		*54*	
*Number of person-years*	*13,541*		*483*	
**Nationality**				
French adolescents	1.00		1.00	
European immigrant adolescents	2.06 *	1.04–4.09	2.02 *	1.06–3.84
Non-European immigrant adolescents	2.58 **	1.34–4.95	1.81 ^§^	0.92–3.57
*All immigrants combined*	*2.31* ***	*1.42–3.76*	*1.92* **	*1.18–3.10*
Girls	1.60 **	1.17–219	1.74 ***	1.25–2.41
Age (year)	1.06	0.94–1.20	1.01	0.88–1.16
**Socioeconomic factors**				
Family structure				
Intact	1.00		1.00	
Divorced or separated parents and reconstructed family	2.72 ***	1.94–3.81	2.38 ***	1.68–3.38
Single parent and other types	1.53 ^§^	0.93–2.51	2.26 ***	1.45–3.52
Father’s occupation				
Managers, professionals, and intermediate professionals	1.00		1.00	
Craftsmen, tradesmen, and firm heads	1.65 *	1.06–2.54	1.50 ^§^	0.96–2.33
Service workers and clerks	1.38	0.76–2.50	1.33	0.73–2.43
Manual workers and other occupations	1.75 **	1.17–2.63	1.43 ^§^	0.94–2.17
Not working	1.75 ^§^	0.97–3.16	1.83 *	1.05–3.20
Low parents’ education (<university)	1.37 *	1.01–1.88	1.46 *	1.06–2.01
Insufficient family income	2.29 ***	1.62–3.25	2.24 ***	1.60–3.05
**School, behavior, and health-related difficulties**				
Grade repetition	1.51 *	1.00–2.31	1.92 **	1.21–3.04
Alcohol consumption	2.95 ***	2.11–4.10	2.64 ***	1.84–3.78
Tobacco consumption	6.07 ***	4.16–8.86	7.73 ***	5.00–11.9
Cannabis consumption	4.10 ***	2.48–6.77	3.38 *	1.22–9.39
Hard-drug consumption	8.54 ***	4.54–16.1	8.13 ***	2.97–22.3
Depressive symptoms	7.51 ***	5.25–10.7	11.88 ***	7.96–17.7
Victim of physical and verbal violence	2.40 ***	1.71–3.36	2.48 ***	1.64–3.76
Victim of sexual abuse	9.52 ***	5.47–16.5	7.22 ***	3.78–13.8

* *p* < 0.05; ** *p* < 0.01; *** *p* < 0.001; **^§^** *p* < 0.10 (close to significance); Various school, behavior, and health-related difficulties were considered only when they started before SA.

**Table 4:** Suicide behaviors for immigrant adolescents vs. French adolescents, and contribution of covariates: odds ratio (OR) or hazard ratio (HR) and 95% confidence interval (CI).

Logistic or Cox Regression Models	Suicide Ideation in the Last 12 Months (SI)			Lifetime Suicide Attempts (SA)		
	OR	95% CI	% ^a^	HR	95% CI	% ^a^
*Number of subjects*	*1559*					
*Number of person-years*				*14,530*		
Odds ratio or hazard ratio adjusted for gender and age (gaOR or gaHR)	2.31 ***	1.42–3.76	100	1.92 **	1.18–3.10	100
With further adjustment for socioeconomic factors ^b^	1.95 **	1.18–3.24	27	1.55 ^§^	0.95–2.53	40
With further adjustment for school, behavior, and health-related difficulties ^b^	1.99 *	1.11–3.54	24	1.14	0.68–1.91	85

* *p* < 0.05; ** *p* < 0.01; *** *p* < 0.001; **^§^** *p* < 0.10 (close to significance); **^a^** Reduction in OR/HR computed with the following formula: (gaOR − OR_extended model_)/(gaOR − 1) or (gaHR − HR_extended model_)/(gaHR − 1); **^b^** See Table 3.
